# Posttranscriptional regulation of gene expression—adding another layer of complexity to the DNA damage response

**DOI:** 10.3389/fgene.2012.00159

**Published:** 2012-08-25

**Authors:** Jorge Boucas, Arina Riabinska, Mladen Jokic, Grit S. Herter-Sprie, Shuhua Chen, Katja Höpker, H. Christian Reinhardt

**Affiliations:** ^1^Division of Hematology and Oncology, Center for Internal Medicine, University Hospital of CologneCologne, Germany; ^2^Division of Molecular Medicine, Rudjer Boskovic InstituteZagreb, Croatia; ^3^Division of Nephrology, Center for Internal Medicine, University Hospital of CologneCologne, Germany

**Keywords:** MAPKAP-kinase 2, HuR, hnRNP A0, TIAR, PARN, DNA damage response, cell cycle checkpoint

## Abstract

In response to DNA damage, cells activate a complex, kinase-based signaling network to arrest the cell cycle and allow time for DNA repair, or, if the extend of damage is beyond repair capacity, induce apoptosis. This signaling network, which is collectively referred to as the DNA damage response (DDR), is primarily thought to consist of two components—a rapid phosphorylation-driven signaling cascade that results in immediate inhibition of Cdk/cyclin complexes and a delayed transcriptional response that promotes a prolonged cell cycle arrest through the induction of Cdk inhibitors, such as p21. In recent years a third layer of complexity has emerged that involves potent posttranscriptional regulatory mechanisms that control the cellular response to DNA damage. Although much has been written on the relevance of the DDR in cancer and on the post-transcriptional role of microRNAs (miRs) in cancer, the post-transcriptional regulation of the DDR by non-coding RNAs and RNA-binding proteins (RBPs) still remains elusive in large parts. Here, we review the recent developments in this exciting new area of research in the cellular response to genotoxic stress. We put specific emphasis on the role of RBPs and the control of their function through DNA damage-activated protein kinases.

## Cells activate a complex signaling network in response to DNA damage

All life on earth must resist a constant assault on its genomic integrity by various endogenous and exogenous sources. Stalled replication forks or incomplete DNA replication during S-phase, and a plethora of different DNA lesions, such as those ubiquitously induced by UV, ionizing radiation (IR), or reactive oxygen species, as well as those intentionally provoked by treatment with chemotherapeutic agents, or radiation therapy used in cancer patients, activate a complex, kinase-based signaling network, which is collectively referred to as the DNA damage response (DDR). Activation of the DDR network through genotoxic lesions triggers signal transduction cascades to activate cell cycle checkpoints, which prevent further progression through the cell cycle as long as the lesions persist (Jackson and Bartek, 2009).

The DDR can be subdivided into two major kinase signaling branches: the ATM pathway, acting through the downstream effector kinase Chk2 and the proximal DDR kinase ATR, acting through Chk1. Some crosstalk exists between the ATM/Chk2 and ATR/Chk1 pathways, particularly when signaling through one pathway is partially or totally deficient (Kastan and Lim, 2000; Abraham, 2001; Shiloh, 2001, 2003; Bartek and Lukas, 2003). Normally however, the pathways appear to have distinct functions with only partial functional overlap in response to particular forms of DNA damage, especially at later stages in the cell cycle (Jazayeri et al., 2006). Different types of genotoxic stress are preferentially channeled through one or the other of these two pathways. The ATM/Chk2 pathway is activated primarily in response to DNA double strand breaks (DSBs), such as those formed by IR or topoisomerase-2 inhibitors, such as etoposide or doxorubicin, while the ATR/Chk1 pathway is activated by bulky DNA lesions induced by UV and in response to replication fork collapse during S-phase (Zhou and Elledge, 2000; Abraham, 2001).

A major target of both the ATM/Chk2 and the ATR/Chk1 branch of the DDR are members of the Cdc25 family of dual specificity phosphatases. Phosphorylation-dependent inhibition of Cdc25 prevents activation of the Cdk-cyclin complexes that mediate transition from G_1_ into S-phase, progression through S-phase and mitotic entry, thus establishing G_1_, intra-S-phase, and G_2_/M cell cycle checkpoints (Donzelli and Draetta, 2003; Rudolph, 2007). Cdc25A is required for activation of Cdk2-Cyclin E and A complexes that govern S-phase entry and progression. Chk1-mediated phosphorylation of Cdc25A creates a phosphodegron motif, resulting in SCF^β−TrCP^-dependent ubiquitination and subsequent proteasomal degradation, as the major mechanism of inhibition (Jin et al., 2003). Cdc25B and C are required for activation of Cdk1-cyclin B complexes mediating mitotic entry. Upon DNA damage Chk1 and 2 phosphorylate Cdc25B and C, creating phosphoepitopes that are recognized and bound by phosphopeptide-binding 14-3-3 proteins (Donzelli and Draetta, 2003; Harper and Elledge, 2007). 14-3-3 serves as a molecular chauffeur resulting in cytoplasmic translocation and sequestration of the complexes, preventing Cdc25B/C from activating Cdk1-cyclin B complexes.

We and others have recently identified a third cell cycle checkpoint effector kinase pathway that is governed by p38α/β-dependent activation of MK2 (Bulavin et al., 2001; Manke et al., 2005; Raman et al., 2007; Reinhardt et al., 2007, 2010; Reinhardt and Yaffe, 2009). This pathway is activated in response to UV and the commonly used chemotherapeutic drugs cisplatin, camptothecin and doxorubicin (Manke et al., 2005; Raman et al., 2007; Reinhardt et al., 2007). We showed that ATM and ATR are required to activate the p38/MK2 module after doxorubicin and cisplatin (Reinhardt et al., 2007). In a series of experiments, we showed that MK2 functions as a downstream checkpoint effector kinase that is critical for cellular survival following DNA damage, specifically in cells and tumors that had lost the prominent tumor suppressor p53 (Reinhardt et al., 2007, 2009; Reinhardt and Yaffe, 2009). MK2 is required to prevent G_1_/S, intra-S phase and G_2_/M transition after cisplatin and doxorubicin in p53-deficient cells (Reinhardt et al., 2007). Intriguingly, MK2 appears to operate in a pathway that is redundant with, but independent of Chk1 (Manke et al., 2005; Reinhardt et al., 2007). Using oriented peptide library screening (OPLS), we determined the amino acid specificity for MK2 phosphorylation and found that it is identical to the optimal sequences selected by the checkpoint kinases Chk1 and Chk2 (Manke et al., 2005; Reinhardt et al., 2007). This finding suggested that all three kinases might share a pool of common substrates. Indeed, we could show that MK2 directly phosphorylates Cdc25A and is required for its DNA damage-dependent degradation, resulting in a G_1_/S arrest after cisplatin and UV (Manke et al., 2005; Reinhardt et al., 2007). In response to doxorubicin, MK2 phosphorylates Cdc25B and C on known Chk1 sites, generating functional 14-3-3 binding sites and resulting in a G_2_/M arrest (Reinhardt et al., 2007). These results suggest that cells lacking a functional p53 response recruit a general stress response network—p38/MK2—to arrest the cell cycle after genotoxic stress. More importantly, this requirement for the p38/MK2 network in p53-deficient tumors, rationalizes the use of MK2 inhibitors as chemosensitizing agents that are based on the synthetic lethal interaction between the corresponding genes *TP53* and *MAPKAPK2* (Reinhardt et al., 2009).

In addition to the activation of this canonical DDR kinase network, which brings about numerous changes in the cellular signaling circuitry occur as a consequence of posttranslational modifications of proteins functioning within the DDR network through phosphorylation, ubiquitylation or sumoylation (Reinhardt and Yaffe, 2009), the pattern of mRNA expression also undergoes significant changes after DNA damage (Rieger and Chu, 2004; Reinhardt et al., 2011). For instance, human lymphoblastoid cells from healthy adults display up- or down-regulation of thousands of mRNAs following exposure to IR or ultraviolet light (Rieger and Chu, 2004). Furthermore, transcriptome analysis following MMS or IR treatment showed that the expression levels of as much as 20% of genes in budding yeast showed a 2-fold or greater change (Gasch et al., 2001). These profound transcriptome alterations appear counterintuitive at first glance, as *de novo* transcription of genes shortly after the infliction of DNA damage might pose a certain threat. The template DNA strand used for transcription might be damaged, leading to the transcription of potentially mutated RNA. In addition, the transcription process is energy-intensive (synthesis of an RNA molecule with *n* bases requires at least *n* NTP molecules) and relatively time-consuming. Specifically, the temporal component imposes a pivotal risk, if the protein product derived from the transcribed mRNA was rapidly needed for cell cycle arrest, DNA repair or the induction of apoptosis. Perhaps not surprisingly, DNA damage, such as that induced by UV-C irradiation, has been shown to trigger a transient repression of transcriptional activity in eukaryotic cells (Vichi et al., 1997; Rockx et al., 2000). Several molecular mechanisms have been implicated in mediating this DNA damage-induced global repression of transcriptional activity. RNA Pol II becomes hyperphosphorylated in response to genotoxic stress and is thus prevented from entering pre-initiation complexes at promoter sites (Rockx et al., 2000; Svejstrup, 2002). Furthermore, *in vitro* evidence suggests that the TATA-binding protein TBP is sequestered onto damaged DNA, reducing its availability for transcription (Vichi et al., 1997; Svejstrup, 2002). The transcriptional repression that is mediated through these molecular pathways varies depending on the type and intensity of DNA damage and is reverted upon completion of DNA repair (Svejstrup, 2002). However, this DNA damage-induced repression of transcriptional activity immediately poses the question how cells accomplish the DNA damage-induced changes in mRNA expression, which have clearly been demonstrated by numerous groups?

## Posttranscriptional regulation of the DNA damage response

As transcription is globally repressed upon DNA damage, additional mechanisms that regulate protein biosynthesis from pre-existing pools of mRNA become critically important to allow an appropriate cellular DDR. Two posttranscriptional regulatory mechanisms are at play to control protein expression following genotoxic stress: (1) selective mRNA stabilization or decay and (2) regulation of translation. Both of these mechanisms critically hinge on the function of RNA-binding proteins (RBPs) and non-coding RNAs, which modulate mRNA stability, transport and translatability through direct interactions with their client mRNAs. Thus, in addition to a well-studied plethora of posttranslational modifications, including phosphorylation, ubiquitination, methylation, acetylation, and others (Harper and Elledge, 2007; Jackson and Bartek, 2009), posttranscriptional control mechanisms are emerging as a new layer of regulation within the complex DDR signaling network.

Intriguing in this regard is data that emerged from a recent phospho-proteomic screen aiming to identify novel ATM/ATR/DNA-PK substrates. The largest subset of substrates identified in these experiments were proteins linked to RNA and DNA metabolism, and specifically proteins involved in posttranscriptional mRNA regulation (Matsuoka et al., 2007). In addition, gene products responsible for nucleic acid metabolism, particularly those involved in mRNA binding and processing, have recently been identified as the largest subset of “hits” in an RNAi-mediated loss of function screen to identify modulators of DNA damage signaling (Paulsen et al., 2009). Furthermore, data provided by Gorospe and co-workers re-enforced the role of posttranscriptional regulatory circuits in the control of a large fraction of the transcriptome in response to genotoxic stress (Fan et al., 2002). Specifically, cDNA expression arrays were employed to gauge the relative contribution of transcription and mRNA turnover to overall changes in gene expression after a variety of cellular stresses, including UV-C irradiation. In essence, a comparison of cDNA hybridization patterns of newly transcribed mRNAs derived from nuclear run-on assays, and steady state mRNA pools derived from whole cell lysates was performed. These experiments revealed that approximately 50% of the changes in mRNA steady state levels that were observed after cellular stress, were attributable to mRNA turnover (stabilization/decay), while the remaining ~50% were due to altered transcription. Lastly, applying a mass spectrometry-based interactome screen, Yaffe and colleagues identified proteins involved in mRNA splicing and translation as the largest group of molecules interacting with the critical DDR protein 14-3-3 (Wilker et al., 2007). These coinciding observations, observed in very different experimental settings, highlight the potential importance of posttranscriptional regulatory mechanisms in the context of DDR signaling, and strongly argue that the DDR may extend substantially beyond the classical ATM/Chk2 and ATR/Chk1 signaling cascades detailed above.

The first links between the kinase-based canonical DDR and posttranscriptional regulatory mechanisms were established through the study of p21^Cip1/WAF^ mRNA. p21^Cip1/WAF^ is a canonical p53 target gene and is potently induced in response to genotoxic stress (el-Deiry et al., 1993). Not only could Wang et al. show that the RBP HuR (human antigen R or ELAVL1, a member of the *e*mbryonic *l*ethal *a*bnormal *v*ision-*l*ike familiy) formed a ribonucleoprotein (RNP) complex with p21^Cip1/WAF^ mRNA in RKO colorectal carcinoma cells following UV-C irradiation, but also that this complex formation appeared to be critical for p21^Cip1/WAF^ mRNA stabilization following genotoxic stress, as HuR depletion impaired p21^Cip1/WAF^ mRNA induction after UV-C (Wang et al., 2000). Further, the laboratory of A. Nebreda recently showed that p38MAPK induces p21^Cip1/WAF^ mRNA stabilization without significantly affecting transcription of p21^Cip1/WAF^ (Lafarga et al., 2009). p38MAPK-mediated phosphorylation of HuR on Thr-118 in response to IR was shown to be critical for cytoplasmic accumulation of HuR, enhanced binding to the p21^Cip1/WAF^ mRNA and subsequent p21^Cip1/WAF^ mRNA and protein accumulation (Lafarga et al., 2009). Further experiments revealed that the shuttling of HuR between the nucleus and the cytoplasm is tightly regulated by a variety of kinases, including Cdk1, Chk2, and MK2 (Tran et al., 2003; Abdelmohsen et al., 2007; Kim et al., 2008). recently suggested that Chk2, which shares substrate homology with MK2 (Manke et al., 2005), phosphorylates HuR on Ser-88, Ser-100, and Thr-118. This interaction is likely to occur in the nucleus, since Chk2 and HuR could be co-immunoprecipitated only from nuclear extracts (Abdelmohsen et al., 2007). Phosphorylation, particularly on Ser-100 in response to genotoxic H_2_O_2_, decreased the binding affinity of HuR to its target mRNA SIRT1 (Sirtuin 1), resulting in destabilization of SIRT1 mRNA, decreased SIRT1 protein levels and increased sensitivity of WI-38 human diploid fibroblasts to the cytotoxic effects of H_2_O_2_. Mutation of Ser-88 and Thr-118 to Ala reduced SIRT1 mRNA binding even in the absence of H_2_O_2_, suggesting that phosphorylation on these sites actually promotes HuR RNP formation. It is, however, also conceivable that these particular mutations induce conformational changes that preclude effective RNA binding, since these residues are located within the RNA recognition motifs (RRMs) of HuR. Interestingly, treatment of WI-38 cells with H_2_O_2_ revealed that binding of wildtype HuR differed according to the target mRNA: binding of p21^Cip1/WAF^ mRNA was increased, while decreased on SIRT1 and numerous cyclin mRNAs. However, mutation of Ser-100 to Ala generally increased the binding affinity of HuR to all mRNAs tested. These observations suggest that although Chk2 is clearly activated by H_2_O_2_, this activity does not translate into a uniform decrease in HuR binding affinity to its target mRNAs. One could speculate that structural features within the HuR target mRNAs or the recruitment of other RBPs into the HuR RNPs ultimately dictate the affinity of HuR to its target mRNAs. It is also possible that the Chk2 recognition motif in HuR might be masked in certain RNPs, which could preclude Chk2-mediated phosphorylation of Ser-100 in certain RNPs. These questions await further clarification.

As a member of the ELAV-like family of RBPs, HuR has strong binding affinity to mRNAs that contain so-called AU-rich elements (AREs) in their 3′ UTR (Dean et al., 2004). AREs act as potent mRNA destabilizing elements that target mRNA for rapid deadenylation (Chen and Shyu, 1994; Xu et al., 1997; Wilson and Treisman, 1988). AREs can be subdivided into three classes: class I and II AREs contain copies of an AUUUA pentameric repeat, called Shaw-Kamen motif (Shaw and Kamen, 1986). Class I AREs contain 1–3 scattered Shaw-Kamen motifs in the 3′ UTR, class II AREs contain multiple, partially overlapping AREs in their 3′ UTR, and class III AREs commonly lack the AUUUA pentamer, but are enriched for U-rich sequence stretches (Dean et al., 2004).

Nagamine and colleagues (Tran et al., 2003) showed that HuR binds and stabilizes the urokinase plasminogen activator (uPA) mRNA in an ARE-dependent manner. The authors went on to show that overexpression of constitutively active MK2 resulted in stabilization of ARE-containing reporter mRNAs. This effect correlated with an MK2-dependent cytoplasmic accumulation of HuR. Furthermore, treatment with H_2_O_2_, a known MK2 activating stimulus, also resulted in cytoplasmic HuR accumulation. The authors demonstrated that increased binding of HuR to ARE-containing uPA mRNA and stabilization of an ARE-containing reporter mRNA in response to H_2_O_2_ depended on MK2 acting downstream of p38MAPK. However, no evidence suggesting that MK2 directly phosphorylates HuR in this system was presented in this study.

In contrast to the molecular effect of p38MAPK, Chk2, and MK2, Cdk1-mediated HuR phosphorylation on Ser-202 was recently shown to sequester HuR in the nucleus (Kim et al., 2008). Cdk1 inhibition promoted a cytoplasmic accumulation of HuR, while a predominately nuclear localization of HuR was observed under conditions of high Cdk1 activity. Furthermore, a Ser-202 to Ala mutant form of HuR was located primarily in the cytoplasm, while phospho-Ser-202 HuR could be detected almost exclusively in the nucleus. Kim et al. further showed that Cdk1-dependent Ser-202 phosphorylation of HuR was essential for 14-3-3θ binding to HuR. However, it was never formally demonstrated that the phosphopeptide-binding protein 14-3-3θ directly binds a phosphoepitope surrounding Ser-202.

Among the known DDR kinases, the p38MAPK/MK2 signaling complex probably has the strongest ties to posttranscriptional control of gene expression. Anderson and colleagues characterized the MK2-mediated regulation of the zinc finger protein Tristetraprolin (TTP), which had been shown to bind and destabilize ARE-containing mRNAs such as TNFα (Stoecklin et al., 2004). ARE-containing mRNAs are unstable under normal conditions and are stabilized in response to various cellular stressors, such as UV, lipopolysaccharides (LPS), or arsenite (Kedersha and Anderson, 2002). In their experiments, Anderson and colleagues showed that MK2-mediated phosphorylation of TTP on Ser-52 and Ser-178 in response to arsenite generated a phosphoepitope that was subsequently engaged by 14-3-3 (Stoecklin et al., 2004). TTP binds to ARE-containing target mRNAs and directs them to exosome-dependent degradation. TTP:14-3-3 complex formation resulted in exclusion from stress granules (SGs) and inhibition of TTP-dependent degradation of ARE-containing β-globin reporter mRNA. SGs are the morphological correlate of an abrupt, stress-induced translational arrest resulting in rapid polyribosome disassembly (Kedersha and Anderson, 2002). These cytoplasmic granules consist of a number of proteins involved in RNA metabolism, as well as stalled initiation complexes, which are bound to numerous mRNAs (Anderson and Kedersha, 2006). The mRNA molecules from disassembled, stalled polyribosomes are sorted into SGs where the fate of each individual messenger is determined by RBPs that either promote RNA stabilization or decay (Kedersha and Anderson, 2002; Kedersha et al., 2005). SG proteins, such as TIA-1 and HuR, bind to ARE-containing mRNAs, and control their stability and translation (Anderson and Kedersha, 2002, 2006; Kedersha and Anderson, 2002; Kedersha et al., 2005). As an alternative mechanism to TTP:14-3-3 complex formation, it could be shown that phosphorylation of TTP by MK2 blocks mRNA decay by inhibiting the recruitment of the CCR4-CAF1 deadenylase complex (Marchese et al., 2010).

Like TTP, BRF1, subunit of the RNA polymerase III, is an ARE-binding protein that has recently been shown to be a direct substrate of MK2. Phosphorylation of BRF1 on four distinct residues (Ser-54, Ser-92, Ser-203, and an unidentified site in the C-terminus) reduced the ability of BRF1 to promote ARE-mediated decay. However, the mechanistic details of this effect remain somewhat unclear (Maitra et al., 2008).

Besides TTP and BRF1, which promote ARE-mediated decay, MK2 has also been shown to directly phosphorylate hnRNP A0, a protein that specifically interacts with ARE-containing mRNAs, exerting a stabilizing effect on its RNA targets. Rousseau et al. (2002) identified hnRNP A0 (heterogeneous nuclear RNP A0) as a protein with binding affinity for the AREs in the 3′ UTR of TNFα in macrophage lysates. They further showed that MK2 phosphorylates hnRNP A0 on Ser-84 following LPS treatment. Pharmacological inhibition of p38MAPK abrogated hnRNP A0 binding to its MIP-2 (macrophage inflammatory protein 2) client mRNA and impaired MIP-2 mRNA stability and protein induction. Together these findings suggest that MK2-dependent phosphorylation of hnRNP A0 is required for mRNA binding and stabilization.

A number of other RBPs have been identified as MK2 substrates *in vitro*, however, the functional relevance of these phosphorylation events remains elusive and awaits further investigation. For example, Bollig and colleagues identified PABP1 (Polyadenylate-binding protein 1) as a GM-CSF (Granulocyte macrophage colony-stimulating factor) ARE-binding protein, which can be efficiently phosphorylated by MK2 *in vivo* (Bollig et al., 2003). Whether this phosphorylation takes place *in vivo* and what influence it might have on GM-CSF mRNA stability or translation remains unclear.

Although, defects in RBPs have been associated with a large number of diseases, our current knowledge is largely still restricted to canonical RNA binding domains and target sequences (Lukong et al., 2008; Cooper et al., 2009; Darnell, 2010). However, major progress is currently being made in our understanding of RBP biology, similar to the extensive achievements concerning the role of microRNAs (miRs) in the posttranscriptional regulation of target mRNAs. Considerable accomplishments in this field were obtained from studies devoted to the systematic discovery of structural elements governing stability of mammalian mRNAs, the generation of an atlas of mammalian RBPs and the identification of target RNAs via high-throughput sequencing of cross-linked RNPs after immunoprecipitation (Hafner et al., 2010; Zhang and Darnell, 2011; Castello et al., 2012; Goodarzi et al., 2012).

## MicroRNA-mediated regulation of the DNA damage response

In addition and complementary to regulation of mRNA stability and translation by RBPs, posttranscriptional control is potently exerted by miRs. These recently discovered, yet ubiquitous molecules, 18–24 nucleotides in length, regulate the stability and/or translation of their target mRNAs by forming imperfect Watson-Crick base pairs within the 3′ UTR. By virtue of this interaction, the microRNA recruits a protein complex referred to as miRISC (miRNA-induced silencing complex) that exerts translational repression by a mechanism that is not yet fully understood. Recently, reported data strongly suggests that destabilization of target mRNAs, instead of translational repression, is the predominant mechanism for reduced protein output (Guo et al., 2010). The minimal protein components of miRISC required for microRNA-mediated this repression are Argonaute (AGO; principally AGO2 in mammals and AGO1 in flies) and TNRC6 (trinucleotide repeat containing 6)/GW182 (glycine-tryptophan protein of 182 kDa) (Guo et al., 2010). One, mechanism of microRNA function that has been proposed is the sequestration of their target mRNAs in sub-cellular compartments that prevent their access to the protein synthesis machinery (Cannell et al., 2008). Two, such compartments implicated in microRNA control are SGs and P-bodies (PBs), both related structures acting as sites of triage for repressed mRNA molecules (Cannell et al., 2008; Buchan and Parker, 2009). The notion that SGs may play an important role for the DDR arises from a study by Pothof et al., who showed that UV-induced DNA damage caused a transient localization of AGO2 to SGs and that cells depleted of AGO2 are hypersensitive to UV-irradiation (Pothof et al., 2009). Furthermore, Zeng et al. demonstrated that MK2 can efficiently phosphorylate AGO2 on Ser-387 and this reaction was induced in HEK293T cells over-expressing AGO2 after treatment with sodium arsenite (Zeng et al., 2008), a known activator of the p38MAPK/MK2 pathway. Besides, examining immortalized human non-small cell lung carcinoma cells (NCI-H1299), the group showed that mutation of Ser-387 to alanine or pharmacological inhibition of p38MAPK reduced arsenite-induced AGO2 recruitment into PBs. This points to a potential role for MK2 signaling in the formation of SGs and PBs.

In addition to the global regulation of the DDR by AGO2, specific miRNAs have been shown to be vitally important for cells to mount a functional DDR. The first example found were the miRNAs of the miR-34 family (miR-34a, miR-34b, and miR-34c), which were simultaneously identified as p53 transcriptional targets by several groups (Chang et al., 2007; Corney et al., 2007; He et al., 2007; Tarasov et al., 2007). These miRNAs appear to act as critical regulators of the DDR by repressing target mRNAs that regulate the cell cycle and apoptosis. Concretely, data presented by Raver-Shapira et al. indicates that inhibition of miR-34a, the most pro-apoptotic member of the miR-34 family, prevented etoposide-induced cell death to the same extent as p53 depletion, suggesting that miR-34a is a potent mediator of p53-mediated apoptosis in this context (Raver-Shapira et al., 2007). The ability of miR-34a to induce apoptosis may be attributable to its ability to repress the anti-apoptotic protein BCL-2 via an interaction in the 3′ UTR of BCL-1 mRNA (Bommer et al., 2007). However, Yamakuchi et al. showed that miR-34a represses SIRT1 through its 3′ UTR and that over-expression of SIRT1 rescued miR-34a-induced apoptosis (Yamakuchi et al., 2008), suggesting that SIRT1 is a functionally important target in that system. In contrast to miR-34a, miR-34b/c do not seem to regulate cell death. Rather, these two highly homologous miRNAs inhibit cell cycle progression in response to DNA damage primarily by repressing the proto-oncogene *C-MYC* in both a p53-dependent and -independent manner (Cannell and Bushell, 2010; Cannell et al., 2010).

Since the initial finding of miR-34, several other miRNAs regulating events both proximal and distal to the initial DNA lesion, have been implicated in the DDR. WIP1 (wild-type p53-induced phosphatase 1), a key phosphatase targeting critical DDR components, such as p53, ATM, and H2AX for dephosphorylation, is also the target of a miRNA (Takekawa et al., 2000; Lu et al., 2005; Shreeram et al., 2006). Specifically, the experiments performed by Zhang et al. (2010) revealed that miR-16, a tumor suppressor miRNA frequently found to be deleted in chronic lymphocytic leukemia (CLL), inhibits WIP1 translation (Calin et al., 2002, 2004; Zhang et al., 2010). According to the authors, WIP1 mRNA levels rapidly increase following DNA damage, while WIP1 protein fails to accumulate. Further, they went on to show that miR-16 levels augment rapidly in response to neocarzinostatin, consequently prevent WIP1 protein accumulation and thus allowing ATM phosphoryaltion to be maintained. At later stages, likely when DNA repair is complete, miR-16 levels decrease, WIP1 protein accumulates again and ATM is dephosphorylated (Zhang et al., 2010). These observations are particularly pertinent in the context of p53 signaling: as well as transcriptionally regulating miR-34, p53 also controls the maturation of certain miRNAs including miR-16 in a posttranscriptional manner (Suzuki et al., 2009). At birth, miRNAs are long primary transcripts termed pri- miRs and are processed in the nucleus by an enzyme called Drosha to become a pre-microRNA (60–70 nucleotides in length). This pre-microRNA is further exported to the cytoplasm and subjected to the RNAse III enzyme Dicer for final processing (18–24 nucleotides). Interestingly, Suzuki et al. demonstrated that p53 forms a complex with Drosha by virtue of an interaction with the DEAD-box RNA helicase p68 (a.k.a DDX5) to augment conversion of pri-miR-16 (amongst others) in a DNA damage-dependent manner (Suzuki et al., 2009). Considering the observation that WIP1 is also a p53 target gene (Fiscella et al., 1997), allows us to hypothesize on the following scenario: p53 transcriptionally induces WIP1 and posttranscriptionally induces miR-16, which limits WIP1 protein production. Upon completion of DNA repair, miR-16 levels decrease and lead to a rise in WIP1 protein and attenuation of ATM signaling. It is tempting to speculate that the association between p53 and p68/DDX5 is regulated by alternative DNA damage signaling pathways to those, which control p53-dependent transcription leading to differential temporal regulation of p53-mediated transcription and miRNA processing.

In addition to the above, downstream events in the DDR signaling cascade are also regulated by miRNAs. By generating cell lines deficient for miR-21, Wang et al. demonstrated that CDC25A is regulated by this miRNA via its 3′ UTR (Wang et al., 2009). The analyses of miR-21 deficient RKO colon cancer cells disclosed increased mitotic entry in response to IR in comparison to their wild-type counterparts. This phenomenon was largely blunted by CDC25A depletion, suggesting that miR-21 regulates a DNA damage induced G_2_/M checkpoint by repressing CDC25A (Wang et al., 2009). It is therefore possible that DNA damage imposes a “double-hit” inhibition on CDC25A function by restraining its translation through miR-21 and promoting its degradation through Chk1/Chk2/MK2 signaling (Reinhardt and Yaffe, 2009). However, it remains enigmatic who are the key players promoting induction of miR-21 in response to DNA damage and whether this executed at the transcriptional or posttranscriptional level.

Very recently, Gorospe and colleagues have uncovered some of the mechanisms mediating miR-519-dependent regulation of the DDR (Abdelmohsen et al., 2012). It was previously known that miR-519 inhibits cell proliferation. This group now identified two prominent subsets of miR-519-regulated mRNAs. First, miR-519 targets mRNAs encoding the DNA maintenance proteins DUT1, EXO1, RPA2, and POLE4 to repress their expression ultimately resulting in increased DNA damage and upregulation of CDKN1A^p21^. The second group of target mRNAs encoded proteins involved in calcium homeostasis, such as, ATP2C1 and ORAI1. Downregulation of these mRNAs raised cytosolic calcium levels, further increasing p21 levels. Together these alterations produced an autophagic phenotype in various cell lines.

Although, the majority of studies regarding non-coding RNA has focused on the function of miRNAs, a plethora of non-coding transcripts still awaits to be analyzed for their role in the DDR [for a detailed review on non-coding RNA in diverse human diseases see (Esteller, 2011)]. Recently, more than 1000 large intergenic noncoding RNAs (lincRNAs) have been reported (Khalil et al., 2009). These RNAs are evolutionarily conserved in mammalian genomes and thus presumably function in diverse biological processes (Khalil et al., 2009). Interestingly, lincRNA-p21 (located near the *CDKN1A* gene encoding the p21 protein) is transcriptionally regulated by p53 and was also shown to interact with hnRNP-K, namely by conveying hnRNP-K to the promoter region of p53 target genes, which in turn become transcriptionally repressed (Huarte et al., 2010). This lincRNA-p21:hnRNP-K interaction was observed to be required for proper genomic localization of hnRNP-K at repressed genes and regulation of p53-mediated apoptosis (Huarte et al., 2010).

More recently, Wei and colleagues elegantly illustrated that so-called DSB-induced small RNAs (diRNAs) are transcribed from sense and antisense strands at, or close to the DSB sites in *Arabidopsis* and human cells (Wei et al., 2012). In *Arabidopsis*, the biogenesis of diRNAs required ATR, RNA Pol IV, and Dicer-like proteins. Mutations in these proteins as well as in Pol V prevented efficient DSB repair (Wei et al., 2012). Subsequently, the authors provided evidence that diRNAs are recruited by AGO2 to establish DSB repair in *Arabidopsis*. Furthermore, depletion of Dicer or AGO2 in human cells led to a similar decrease in DSB repair efficiency. The authors propose diRNAs to serve as guiding molecules directing chromatin modifications or the recruitment of protein complexes to DSB sites in order to ultimately facilitate DSB repair (Wei et al., 2012).

## Gadd45α is posttranscriptionally regulated in response to DNA damage

**Figure 1:**
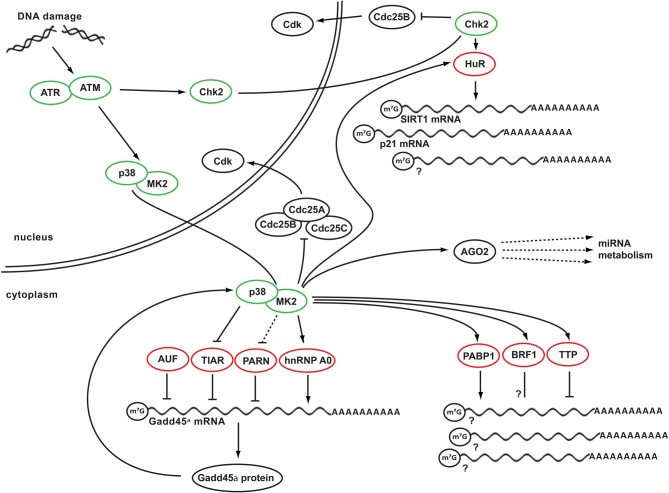
**DDR kinase signaling at the crossroads of cell cycle arrest and posttranscriptional control of RNA stability.** Depicted is a simplified schematic network integrating key DDR kinases and RNA-binding proteins. In response to genotoxic stress, ATM activates its effector kinase Chk2 and the p38MAPK/MK2 kinase complex. Chk2 in turn phosphorylates HuR, promoting its binding to SIRT1 mRNA. Binding of HuR to additional client mRNAs, such as p21 mRNA appears to be regulated by MK2, which also mediates RNA binding of several other RBPs, including PABP1, BRF1, and TTP. In addition, MK2 phosphorylates hnRNP A0, promoting its binding to and stabilization of Gadd45α mRNA. In the absence of DNA damage, Gadd45α mRNA is destabilized and translationally repressed through the RNA-binding proteins PARN, TIAR, and AUF. These RBPs dissociate from the Gadd45a mRNA after genotoxic stress. Gadd45α protein is part of a positive feedback loop that maintains p38/MK2 activity at late times following DNA damage. Prolonged MK2 activity in turn is required to maintain Cdc25B and C in an inactive state sequestered in the cytoplasm. Finally, mRNA of numerous players in DDR signaling is being regulated by miRNAs, which require AGO2 protein to convey their regulation. AGO2 is, in turn, is a phospho-target of MK2. Green circles indicate DNA damage-activated kinases, red circles indicate RNA-binding and metabolizing proteins.

In addition to p21^Cip1/WAF^ mRNA, which has been demonstrated to be posttranscriptionally stabilized after DNA damage, Fornace and colleagues identified Gadd45α mRNA as posttranscriptionally stabilized in response to genotoxic stress (Jackman et al., 1994) (Figure 1). Gadd45α is part of a family of genes consisting of Gadd45α, Gadd45β, and Gadd45γ that is widely expressed in mammalian cells following different stress stimuli. Gadd45α is induced following hypoxia, IR, oxidants, UV, and growth factor withdrawal (Zhan, 2005). Gadd45α has been mechanistically linked to numerous cellular processes, including apoptosis, cell cycle arrest, nucleotide excision repair and repair-mediated DNA demethylation, maintenance of genomic stability and signaling through the p38MAPK, and JNK kinase pathways (Hollander et al., 1999; Wang et al., 1999; Smith et al., 2000; Amundson et al., 2002; Hildesheim et al., 2002; Barreto et al., 2007). Gadd45α expression is rapidly induced after genotoxic stress. This transcriptional activation has initially been thought to be primarily induced by p53 (Kastan et al., 1992). In fact, p53 was the first transcription factor reported to induce Gadd45α transcription and, at least in response to IR, Gadd45α transcription strictly depends on p53 (Kastan et al., 1992). However, it is now clear that additional transcription factors, including WT1, Oct1, NF-YA, FoxO3a, Egr-1, and C/EBPα are also capable of inducing Gadd45α transcription, even in the absence of p53 (Constance et al., 1996; Zhan et al., 1998; Jin et al., 2001; Takahashi et al., 2001; Tran et al., 2002; Hirose et al., 2003; Thyss et al., 2005). For example, we recently showed that Gadd45α was induced in p53-deficient murine embryonic fibroblasts (MEFs) following treatment with doxorubicin (Jiang et al., 2009). In resting cells, Gadd45α transcription appears to be repressed through c-Myc and a repressive complex consisting of ZBRK1 and BRCA1 (Marhin et al., 1997; Amundson et al., 1998; Bush et al., 1998; Zheng et al., 2000; Tan et al., 2004). Interestingly, c-Myc itself is translationally repressed through miR-34c via a highly conserved target-site within the 3′ UTR in response to etoposide-induced DNA damage. While miR-34c can be induced by p53 following genotoxic stress, Cannell et al. (2010) showed that miR-34c expression in p53-deficient cells depends on the p38MAPK/MK2 signaling complex (Cannell et al., 2010). In addition to this elaborate network of transcriptional control, Fornace and colleagues reported as early as 1994 that Gadd45α mRNA is posstranscriptionally stabilized in response to UV or MMS exposure (Jackman et al., 1994). However, the molecular details of this posttranscriptional regulation remained largely obscure. These posttranscriptional regulatory mechanisms might impact on Gadd45α mRNA molecules at different steps of their maturation, from their *de novo* synthesis as pre-mRNA until the eventual degradation or translation. These steps include pre-mRNA splicing and maturation (3′ polyadenylation, 5′ capping), followed by mRNA export to the cytoplasm, sub-cytoplasmic transport, escape from ribonucleolytic cleavage and translation (Mitchell and Tollervey, 2000; Orphanides and Reinberg, 2002; Moore, 2005). Recent studies from Gorospe and co-workers have identified the RBPs AUF1 and TIAR as critical posttranscriptional regulators of Gadd45α mRNA (Lal et al., 2006). Both proteins were found to form RNP complexes through a direct interaction with the 3′ UTR of the Gadd45α mRNA in resting cells. However, when cells were exposed to UV or MMS these RNP complexes rapidly dissociated, which correlated with a substantial increase in Gadd45α mRNA stability an enhanced association of Gadd45α mRNA with actively translating ribosomes and increased Gadd45α protein accumulation (Lal et al., 2006). When Lal et al. examined the molecular mechanisms of Gadd45α repression in resting cells, they found AUF1 to render Gadd45α mRNA unstable while TIAR prevented the association of Gadd45α mRNA with translating polyribosomes. Thus, the combined effect of AUF1 and TIAR is a potent repression of Gadd45α biosynthesis through AUF1-mediated mRNA destabilization and TIAR-dependent translational suppression at resting state. The genotoxic stress-induced dissociation of AUF1 and TIAR from the Gadd45α mRNA represents a mechanism of posttranscriptional derepression resulting in mRNA stabilization and enhanced translation in response to DNA damage. Both of these posttranscriptional regulatory steps were found to be essential for proper induction of Gadd45α protein levels following DNA damage (Lal et al., 2006).

The report by Lal et al. implicated AUF1 and TIAR as RBPs that are critical for the posttranscriptional de-repression of Gadd45α mRNA. However, it remained unclear which molecular mechanisms underlie the DNA damage-induced dissociation of these RBPs from the Gadd45α mRNA. A plausible explanation might be DNA damage-dependent phosphorylation events. Indeed, AUF1 was reported to be a phospho-protein and GSK3β and PKA were subsequently identified as kinases capable of AUF1 phosphorylation *in vivo* (Zhang et al., 1993; Wilson et al., 2003). Nonetheless, whether these phosphorylations occur *in vivo* following genotoxic stress persists to be elusive.

We have recently identified the p38MAPK/MK2 pathway as a critical regulator of RBPs that mediate posttranscriptional stabilization of Gadd45α mRNA in response to genotoxic stress (Reinhardt et al., 2010). In analyzing the molecular details of MK2 function in response to DNA damage, we found that MK2 knockdown prevented the accumulation of Gadd45α mRNA and protein in response to adriamycin. We identified the known MK2 substrate hnRNP A0 as a novel Gadd45α mRNA-binding protein (Reinhardt et al., 2010). MK2-mediated phosphorylation of hnRNP A0 on Ser-84 following DNA damage was required for the formation of hnRNP A0:Gadd45α mRNA RNP complexes and overexpression of a non-phosphorylatable hnRNP A0 on Ser-84 to Ala mutant prevented Gadd45α mRNA and protein accumulation in response to adriamycin (Reinhardt et al., 2010). These data suggest that MK2-dependent phosphorylation of hnRNP A0 is critical for the formation of hnRNP A0:Gadd45α mRNA RNP complexes, which in turn appears to be essential for the posttranscriptional stabilization of Gadd45α mRNA. In addition, we found that MK2 phosphorylates *P*oly-(*A*) *r*ibo*n*uclease (PARN) on Ser-557 in response to adriamycin (Reinhardt et al., 2010). Two major pathways of mRNA degradation exist in eukaryotes. In both cases, shortening of the poly(A) tail is the first, time-limiting, step. Three distinct protein complexes—Pan2/Pan3, or PAN complex; PARN; and the Ccr4/Pop2 complex—govern this deadenylation. After deadenylation, degradation occurs in 3′–5′ direction through the RNase-containing exosome complex. In an independent pathway, deadenylation is followed by removal of the 7-methyl-guanosine cap of mRNAs and then proceeds in the 5′–3′ direction. The mechanisms of mRNA turnover have been reviewed recently (Meyer et al., 2004). We found PARN phosphorylation on Ser-557 to be critical for prolonged Gadd45α mRNA and protein expression after adriamycin (Reinhardt et al., 2010). However, the molecular details of this apparent inhibition of Gadd45α mRNA degradation remain somewhat unclear. Despite our best efforts, we failed to observe any changes in PARN activity or RNA binding affinity following MK2-mediated phosphorylation on Ser-557 (Schmedding, Reinhardt, Yaffe unpublished). In addition to these MK2-mediated posttranscriptional mechanisms of Gadd45α mRNA stabilization, we confirmed that TIAR dissociates from the Gadd45α mRNA in response to genotoxic stress (Reinhardt et al., 2010). Furthermore, we could show that p38MAPK directly phosphorylates TIAR after adriamycin exposure, both *in vitro* and *in vivo* [(Reinhardt et al., 2010) and Morandell, Reinhardt, Yaffe unpublished]. Pretreatment of cells with the p38α/β-specific inhibitor SB203580 completely prevented the adriamycin-mediated dissociation of TIAR:Gadd45α mRNA RNP complexes. Thus, we have identified three novel mechanisms of posttranscriptional Gadd45α mRNA control. We identified hnRNP A0 as a critical MK2-dependent posttranscriptional inducer of Gadd45α mRNA. In addition to AUF and TIAR, which have been described as posttranscriptional repressors of Gadd45α mRNA, we have identified PARN as a further molecule that appears to be involved in Gadd45α mRNA repression at resting state. Lastly, we could show that the DNA damage-induced dissociation of the TIAR:Gadd45α mRNA RNP complex depends on p38MAPK-mediated TIAR phosphorylation.

In additional experiments we could confirm data provided by Bulavin et al. showing that Gadd45α interacts with p38MAPK (Bulavin et al., 2003). Bulavin et al. further showed that Gadd45α is critical for H-ras^V12^-induced activation of p38MAPK. We made a similar observation in response to adriamycin-invoked genotoxic stress. RNAi-mediated knockdown of Gadd45α prevented the prolonged phosphorylation and activation of MK2, likely through a lack of p38MAPK activity. MK2 remained active in control cells for at least 30 h. However, MK2 activity dropped precipitously after ~24h in Gadd45α-depleted cells. These data suggest that the initial activation of MK2 after genotoxic stress does not depend on Gadd45α, but subsequent p38MAPK/MK2-dependent stabilization of Gadd45α, through phosphorylation of TIAR, PARN, and hnRNP A0, becomes essential for maintaining MK2 activity at late times. Further experiments showed that particularly this late MK2 activity was critical to maintain checkpoint control after genotoxic stress invoked by doxorubicin through a mechanism involving Cdc25B/C inactivation. Members of the Cdc25 family of dual-specificity phosphatases are phosphorylated by the checkpoint effector kinases Chk1 and MK2 in response to DNA damage. We and others previously showed that the cell cycle arresting checkpoint function of MK2 is mediated through MK2-dependent Cdc25B/C phosphorylation and subsequent cytoplasmic sequestration (Lopez-Aviles et al., 2005; Manke et al., 2005; Reinhardt et al., 2007).

We note that MK2 and its activating kinase p38MAPK form a tight nuclear complex in resting cells (Ben-Levy et al., 1995, 1998; ter Haar et al., 2007). MK2 contains a nuclear localization signal (NLS) and a nuclear export signal (NES) located at the C-terminus. At resting state, the NES is masked by a direct intramolecular interaction (ter Haar et al., 2007). Following p38-mediated activating phosphorylation of MK2 on Thr-334, this interaction is relieved and the NES becomes exposed, resulting in cytoplasmic translocation of the p38MAPK/MK2 complex (Ben-Levy et al., 1995, 1998; ter Haar et al., 2007). We could show that MK2 rapidly leaves the nucleus in response to DNA damage via a Crm1-dependent nuclear export mechanism (Reinhardt et al., 2010). Thus, we hypothesized that late cytoplasmic MK2 activity might be required to maintain Cdc25B/C sequestered in the cytoplasm in the context of active cell cycle checkpoints. We have hence used live cell imaging to follow the subcellular distribution of Cdc25B and C after genotoxic stress in control cells or cells that were depleted of either Chk1 or MK2. Cytoplasmic accumulation of GFP-tagged Cdc25B/C was used as a readout for active checkpoint signaling. These experiments revealed that adriamycin exposure induces a robust cell cycle checkpoint in control cells that is relieved after ~30 h and is followed by a cytologically normal mitotic cell division. Cdc25B/C was maintained in the cytoplasm until cells entered mitosis. In contrast, Chk1 depletion resulted in premature nuclear re-entry of Cdc25B/C after ~15 h, followed by catastrophic mitotic cell division resulting in apoptosis. We observed a similar phenotype in MK2-depleted cells. However, Cdc25B/C nuclear re-entry did not occur until ~23 h following doxorubicin. Intriguingly in this regard is the observation that this time corresponds perfectly to the time when MK2 activity returned to baseline levels in Gadd45α-depleted cells that were treated with doxorubicin. These data strongly suggest that the positive feedback loop involving MK2-dependent stabilization of Gadd45α, and Gadd45α-dependent maintenance of MK2 activity, are essential for prolonged cell cycle arrest through cytoplasmic Cdc25B/C sequestration in response to adriamycin. Together, these data suggest that a feed forward loop consisting of p38, MK2, and Gadd45α is critical to provide time to recover from adriamycin-induced genotoxic insults before entering the next mitotic cell division.

## Concluding remarks

Posttranscriptional control of gene expression has recently moved into the focus of scientists working in various areas of life sciences. This is owed to the discovery of miRNA-mediated gene silencing mechanisms and the uncovering and characterization of a number of RBPs that are involved in the stabilization and translatability of mRNAs. The DDR network has classically been regarded as consisting of a fast-acting kinase signaling branch, leading to the rapid inactivation of Cdk-cyclin complexes and a delayed transcriptional response, resulting in the transactivation of genes encoding for Cdk inhibitors, such a p21. As a consequence of numerous recent discoveries, a clearer picture is emerging stressing the molecular mechanisms involved in posttranscriptional control of gene expression and expanding the complex DDR signaling network with a third layer. These recent reports strongly suggest that cells employ complex regulatory circuits impacting on transcript stability and translatability in response to genotoxic stress. The major challenges in this emerging area of research in the field of DNA damage signaling are the identification of transcripts that are posttranscriptionally regulated and the identification and functional characterization of proteins that mediate this posttranscriptional control. New technologies, such as, genome-wide RNAi screening and next generation sequencing of cell lines and primary tumor material will promote the identification and functional characterization of non-coding RNAs, RBP, and regulatory RNA sequences involved in the initiation, maintenance and termination of DDR signaling in human tissue.

### Conflict of interest statement

The authors declare that the research was conducted in the absence of any commercial or financial relationships that could be construed as a potential conflict of interest.
